# A survey of blood conservation methods in clinical practice in some urban south-eastern government hospitals in Nigeria

**DOI:** 10.4103/0973-6247.75984

**Published:** 2011-01

**Authors:** A. O. Amucheazi, V. O. Ajuzeiogu, H. A. Ezike, M. C. Odiakosa, O. M. Nwoke, E. Onyia

**Affiliations:** *Department of Anaesthesia, University of Nigeria Teaching Hospital, Ituku-Ozalla, Nigeria*; 1 *Department of Anaesthesia, National Orthopaedic Hospital Enugu, Enugu, Nigeria*; 2 *Department of Anaesthesia, ENUGU State University of Technology Teaching Hospital, Enugu, Nigeria*

**Keywords:** Auotologous blood, blood conservation, minimize homologous transfusion

## Abstract

**General Objective:**

To assess the practice of blood conservation.

**Specific Objectives:**

To determine the methods of blood conservation in use, to assess the lower limit for hemoglobin for elective procedures, to determine transfusion trigger point in practice, to find out limitations in practice and ways to improve clinical practice.

**Materials and Methods:**

This was conducted in February 2009. Self-administered questionnaires were distributed among the surgeons and anesthetists in practice at the University of Nigeria Teaching Hospital, Enugu State University Teaching Hospital, Ebonyi State University Teaching Hospital and National Orthopaedic Hospital, Enugu. The data gathered was analyzed using the SPSS software.

**Results:**

: Of participants who agreed to fill the questionnaires, more than 50% were males. The most prevalent specialty was general surgery (24.2%), followed by orthopedics (22.6%), obstetrics and gynecology (20.7%), and anesthesia (17.7%). The lowest hemoglobin limit before the patient was allowed into the theatre for elective procedures was 10 g/dl while individual transfusion trigger points ranged from hemoglobin of 6 to 10 g/dl. Majority of the doctors would avoid homologous blood transfusion in order to avoid transfusion-related diseases and reaction. Regarding knowledge of blood conservation methods and means of avoiding homologous blood, the use of diathermy was highest (12.33%), followed by preoperative blood donation (11.87%), use of hematinics (10.96%), and tourniquet 10.5%. Also, in practice, diathermy was the most frequently used (18.69%), followed by preoperative blood donation (16.16%), use of tourniquet (15.15%), while the Ovadje cell saver was least with 0.01%. Suggestions from respondents on the ways of limiting transfusion-related problems included optimization of patients (24.5%), improvement of standard of living (17.7%), and personnel training (13.3%).

**Conclusion:**

There is an agreement with the global trend geared toward minimizing the use of homologous blood by doctors in these hospitals. However, our practice must continually be refined by continuing medical education in order to keep everyone informed of changes in practice. The Government must improve the quality of service by the provision of unavailable infrastructure.

## Introduction

During the perioperative period, substantial blood loss may be common and may render patients anemic.[1] Transfusion of allogenic blood may be a life-saver intraoperatively but is now considered undesirable by healthcare professionals due to concerns about transmission of viral infections.[2 3] The importance of blood conservation in order to limit the use of homologous blood is well accepted worldwide.[4] A successful program to this end should involve an integrated approach that begins with preoperative patient preparation, acute normovolemic hemodilution, improvement of surgical skills, as well as ways of limiting blood loss. This would include the use of diathermy, hypotensive anesthesia among others.[5]

This study was designed to examine the practice of blood conservation among doctors in some government-owned tertiary hospitals. Developing nations may lack manpower as well as infrastructure; thus, areas of deficiency may need to be outlined. As blood transfusion practices are being re-examined and redefined, are we doing enough for our patients?

## Materials and Methods

Self-administered questionnaires were distributed among the surgeons and anesthetists in practice at the University of Nigeria Teaching Hospital, Ituku-Ozalla, National Orthopaedic Hospital Enugu, Enugu State University of Technology Teaching Hospital, Enugu, and Ebonyi State University Teaching Hospital Abakaliki. The returned questionnaires were subjected to analysis using the SPSS software.

## Results

**Figure 1:**
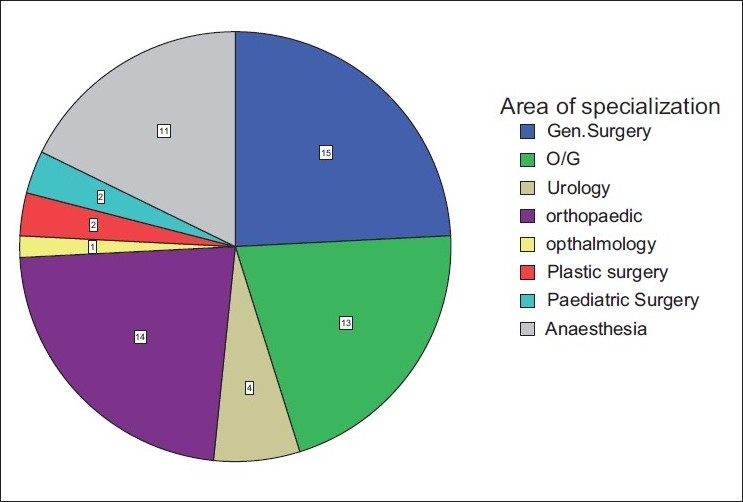
Area of specialization

**Figure 2:**
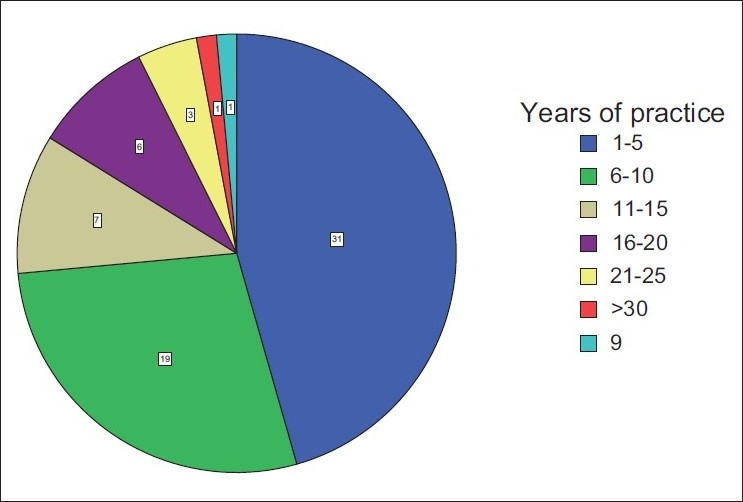
Years of practice

**Figure 3:**
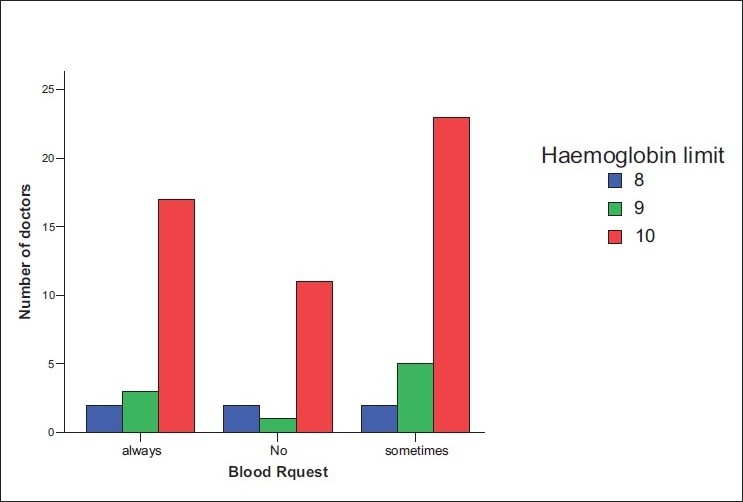
Graph of hemoglobin limit before scheduling for surgery versus frequency of request for blood

**Figure 4:**
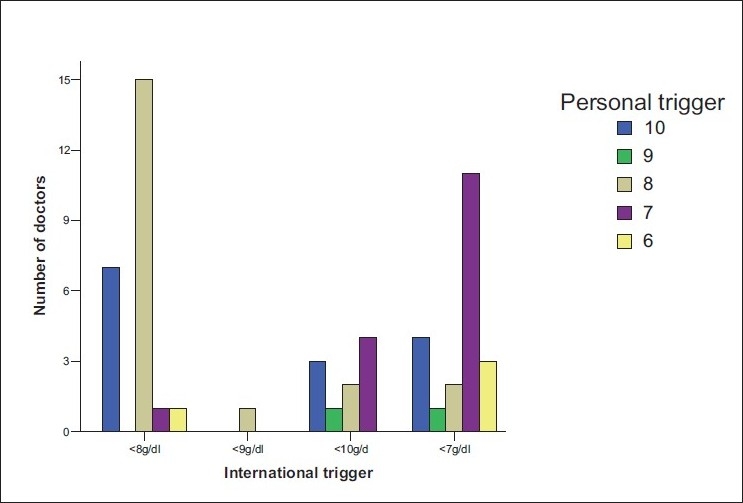
Knowledge of international versus personal trigger level

Sixty-eight participants who were approached agreed to fill the questionnaires. The age range of the respondents was 20–69 and 30–39 years age group was predominant. More than 50% were males. Most of the respondents specialized in general surgery (24.2%), followed by orthopedics (22.6%), obstetrics and gynecology (20.7%), and anesthesia (17.7%) [Figure 1]. Thirty-one (45.6%) doctors had practiced for 1–5 years while nine (13.2%) had put in 16–25 years in practice [Figure 2]. A larger percentage of the respondents were from the University of Nigeria Teaching Hospital (43.3%), followed by Ebonyi State University Teaching Hospital (26.1%), the National Orthopaedic Hospital Enugu (17.4%), and the least number was from the Enugu State University of Technology Teaching Hospital (13.2%). The lowest hemoglobin limit before the patient was allowed into the theater was commonly 10 g/dl [Figure 3]. Fewer doctors would allow a patient of hemoglobin level of 8 g/dl into the theater for elective procedures. Requests for blood before surgery continue to feature, though some respondents would not request for blood to be available prior to surgery. During this study, it was discovered that not all the respondents knew the internationally accepted trigger before a patient should be transfused [Figure 4]. Even among those who knew the internationally accepted trigger point, not all practiced it as individual transfusion trigger points ranged from hemoglobin of 6–10 g/dl.

**Figure 5:**
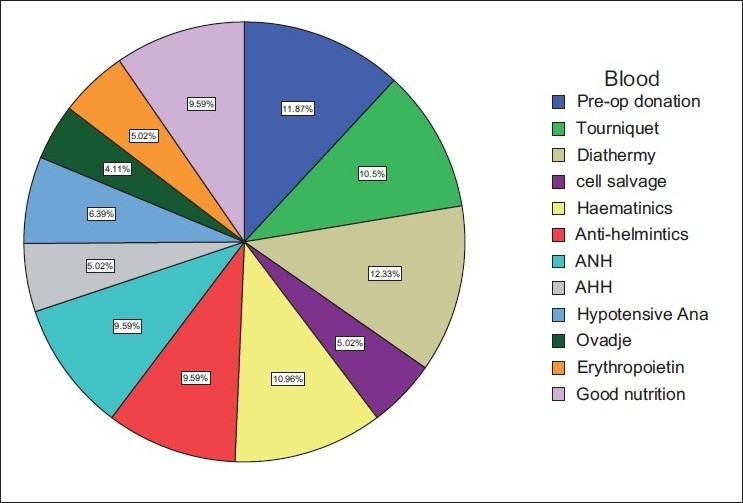
Pie chart showing knowledge of means of blood conservation and limiting of homologous transfusion

**Figure 6:**
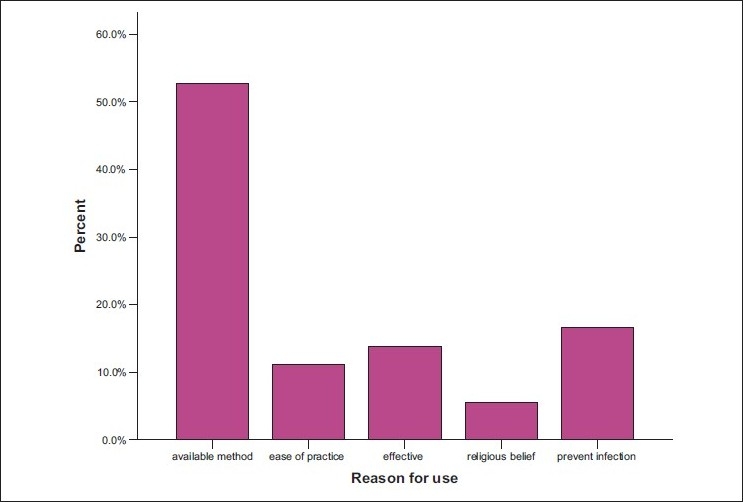
Bar chart showing reason for use of blood conservation method

Majority of the doctors would avoid homologous blood transfusion in order to avoid transfusion-related diseases and reaction. On the safety of the screening of blood, the larger groups were not satisfied with it but had no choice but to accept the method available. The respondents would, however, check the screening label just before transfusion or request for rescreening the morning of or the day before the scheduled surgery. Concerning the knowledge of various methods of blood conservation and limiting homologous blood transfusion, the use of diathermy accounted mostly 12.33% followed by preoperative blood donation 11.87%, use of hematinics 10.96%, and tourniquet 10.5% [Figure 5]. The use of antihelminthics scored 9.5%, acute normovolemic hemodilution had 9.59%, good nutrition 9.59%, hypotensive anesthesia 6.39%, cell salvage, erythropoietin, and acute hypervolemic hemodilution 5.02%, while the use of the Ovadje cell saver had 4.11%. However in practice, diathermy was the most frequently used 18.69%, followed by preoperative blood donation 16.16%, use of tourniquet 15.15%, while the Ovadje cell saver was least with 0.01% frequency of use [Figure 6]. Greater than 50% practiced what they did based on the availability of materials, 10% based on ease of practice, 15% on effectiveness, and 20% on the need to prevent infections. Preponderant suggestions from respondents with the aim of limiting transfusion-related problems included optimization of patients (24.5%), improvement of standard of living (17.7%), and personnel training (13.3%).

## Discussion

There is a consensus that we need to minimize homologous blood transfusion during surgery.[4] Concerns about the cost and safety of allogenic blood transfusion include the risk of transferring viral infections and immunosuppression. These mandate that doctors be amenable to change and be cognizant of these changes in practice with the aim of delivering competent care.[6] The study revealed that the greater percentage of the doctors were cognizant of these changes but were hampered by availability of infrastructure.

According to Massenet *et al*., a serious problem of HIV contamination at undetected levels exists.[7] Mbanga *et al*.in their study revealed that 50% of patients who received homologous blood had an unfavorable outcome: 40% of these had febrile reactions, while 19% had urticaria and 14% died from transfusion-related events.[8] Factors mediating against safe blood transfusion practice include lack of knowledge among donorsand inferior quality of blood.[9] Refinements in and new approaches to blood conservation including development of transfusion practice standards and improvement in surgical skills are thus needed.

Techniques for minimizing blood loss should be started preoperatively. Good nutrition, treatment of helminthiasis especially in children, use of hematinics are important. Massenett *et al*. have called for prevention and treatment of anemia from dietary deficiency as have doctors in this study. This is because preoperative optimization of the patient’s hemoglobin level features greatly in limiting blood transfusion.[7] The use of recombinant erythropoietin, epoetin alfa, has also contributed to a reduction in the use of allogenic blood.[10] This unfortunately is not available in our environment.

Patients undergoing elective surgeries can donate their own blood for use especially when the probability for transfusion is high. Preoperative autologous donation is a cost-effective way of preventing homologous transfusion.[11] In a study by Magoha *et al*.,patient deposit of blood was sufficient to meet their needs and saving one patient who needed augmentation with homologous blood.[12] Preoperative donation of blood by patients or close relatives featured prominently in practice

Acute normo/hypervolemic hemodilution can be used to reduce the need for homologous blood. This relies on the premise that reduction in red cell concentration would lead to a reduction in total red cell loss during surgical procedures. In a study by Daves *et al*., hemodilution has been found to be better than cell salvage and is practiced by doctors in our environment.[13]

Blood salvage and reinfusion are widely used where blood loss is appreciable.[14] The associated problems include unavailability of the machine in the centers studied as well as a risk of septic contamination. The Ovadje cell saver, which would have been accessible as a local product, is however not available.

Blood management in surgical procedure should include reduction of perioperative blood loss. This can be obtained by the use of diathermy, tourniquet, hypotensive anesthesia, and antifibrinolytic agents such as aminocaproic acid and desmopressin. Oginni *et al*. have demonstrated the usefulness of tourniquet even among sickle cell patients.[15] The use of diathermy and tourniquet features prominently among the respondents.

## Conclusion

Doctors in the governmental hospitals in the south-eastern region of Nigeria consider it worthwhile pursuing efforts to de-emphasize homologous blood transfusion while emphasizing blood conservation strategies. Informed selection of alternatives based on preoperative assessment of hematological status and estimation of blood loss would enhance best management practices in surgery and anesthesia. We, however, need provision of cell savers by the government especially for surgeries where blood loss is appreciable.

**Table:** THE QUESTIONNAIRE

1	Age					
	20-29	30-39	40-49	50-59	60-69	
2	Sex					
	Male	Female				
3	Area of specialisation:					
	Anaesthesia	O/G	General surgery Urology	.....................................................		
4	Number of years of practice:					
	1-4	5-9	10-14	15-19	20-24	25-29	30>
5	Hospital attached to:				
	University of Nigeria Teaching Hospital, National Orthopaedic Hospital Enugu, Ebonyi State University Teaching Hospital, Enugu State University of Technology Teaching Hospital						
6	What is your haemoglobin limit before patient is allowed to theatre;					
	8	9	10			
7	How often do you request for blood for surgery:					
	always	no	sometimes			
8	The internationally accepted trigger point for blood transfusion is:					
	<8g/dl,	9g/dl	10g/dl			
9	Your personal trigger point for transfusion is:					
	6g/dl,	7g/dl,	8g/dl,	9g/dl,	10g/dl	
10	Would you avoid homologous blood transfusion					
	Yes	No				
11	Why					
	Avoid infection	transfusion reaction				
12	Are you pleased with the blood screening:					
	Yes,	No,	No choice but to accept it			
13	Do you rescreen blood before transfusion:				
	Satisfied with the screening label,	rescreen on morning of surgery,				
	rescreen the day before and the morning of surgery					
14	The methods of blood conservation and limiting of homolgous blood transfusion you know are:					
	a) use of diathermy b) pre-operative blood donation c)haematinics d) tourniquet e) antihelminthics f) acute normovolaemic haemodilution f) good nutrition g) hypotensive anaesthesia h) cell salvage i) erythropoietin j) acute hypervolaemic haemodilution k) Ovadje cell saver											
15	Of these what is your regular practice					
	a) use of diathermy b) pre-operative blood donation c)haematinics d) tourniquet e) antihelminthics f) acute normovolaemic haemodilution f) good nutrition g) hypotensive anaesthesia h) cell salvage i) erythropoietin j) acute hypervolaemic haemodilution k) Ovadje cell saver											
16	Indicate your reason for your choice in 15:					
	a) method available b) easy to practice c) effectice d) religious belief e) need to prevent infection					
17	Suggest ways of limiting transfusion related problems……………………………………					
